# Early life stress leads to sex differences in development of depressive-like outcomes in a mouse model

**DOI:** 10.1038/s41386-018-0195-5

**Published:** 2018-09-06

**Authors:** Haley L. Goodwill, Gabriela Manzano-Nieves, Meghan Gallo, Hye-In Lee, Esther Oyerinde, Thomas Serre, Kevin G. Bath

**Affiliations:** 1 Department of Neuroscience, Brown University, Providence, RI 02912 USA; 2 Department of Cognitive, Linguistic, and Psychological Sciences, Brown University, Providence, RI 02912 USA

**Keywords:** Stress and resilience, Depression

## Abstract

Childhood trauma and neglect influence emotional development and increase the risk for and severity of mental illness. Women have a heightened susceptibility to the effects of early life stress (ELS) and are twice as likely as men to develop debilitating, stress-associated disorders later in life, such as major depressive disorder (MDD). Until now, mouse models of depression have been largely unsuccessful at replicating the diverse symptomatology of this disease and the sex bias in vulnerability. From P4 to P11, a limited bedding model that leads to fragmented maternal care, was used to induce ELS. Early adolescent and young adult mice were tested on an array of assays to test for depressive-like behavior. This included our newly developed automated home cage behavioral recognition system, where the home cage behavior of ELS and control mice could be monitored over a continuous 5–10 day span. ELS females, but not males, exhibited depressive-like behaviors on traditional assays. These effects emerged during adolescence and became more severe in adulthood. Using the novel home cage video monitoring method, we identified robust and continuous markers of depressive-like pathology in ELS females that phenocopy many of the behavioral characteristics of depression in humans. ELS effects on home cage behavior were rapidly rescued by ketamine, a fast-acting antidepressant. Together, these findings highlight that limited bedding ELS (1) produces an early emerging, female-specific depressive phenotype that responds to a fast-acting antidepressant and (2) this model has the potential to inform sex-selective risk for the development of stress-induced mental illness.

## Introduction

Early life stress (ELS) has life-long consequences on neural and behavioral development and is associated with a significant increase in the risk for mental illness, including anxiety and depression [1, 2]. Females are at increased risk for developing stress-associated pathology with a female/male risk ratio of ~2:1 [3–5]. Sex differences extend beyond prevalence rate, and also exist in symptom manifestation, onset, course of illness, and treatment efficacy [6, 7]. While the etiology of depression is complex, a history of ELS is highly predictive of the first onset of disorder [3, 4, 8]. Despite the significant sex disparities in stress-associated pathology, the mechanisms supporting female susceptibility are largely unknown. Animal models represent a fertile testing ground to probe potential mechanisms underlying risk and mental illness in humans [9, 10], but few studies have tested females in ELS models, and even fewer recapitulate the sex bias in risk for negative outcomes.

For an infant, the interaction with its parent is a vital relationship as its nervous system develops. Disrupting this interaction can result in abnormal brain development, growth, and behavior [11–16]. ELS in the form of poor early life care profoundly alters the development of stress-responsive circuitry [17–19], leading to elevated basal stress hormone levels [20–23], and impaired regulation of the hypothalamic-pituitary-adrenal (HPA) axis [18, 24, 25]. Chronic engagement of the stress response can impact the development of circuits underlying emotional regulation and contribute to later development of anxiety and depressive-like outcomes [14, 26, 27]. Additionally, recent work has identified ELS-induced structural and functional changes in reward circuitry centers in the brain that are linked with depression and other mood disorders [28–30], suggesting mechanisms of ELS above and beyond the canonical HPA axis may contribute to depressive behaviors.

**Fig. 1:**
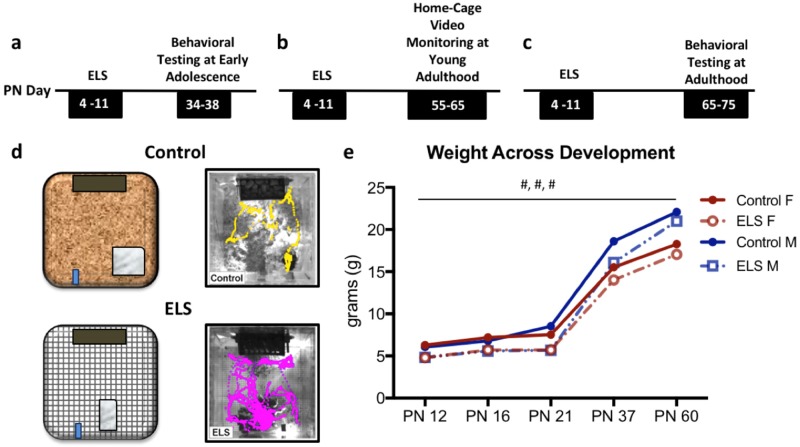
ELS results in fragmented maternal care and altered somatic development in both male and female mice. **a**–**c** Timelines for groups of mice undergoing testing at early adolescence (**a**), young adulthood (**b**), or in adulthood (**c**). **d** A schematic illustration of control and ELS cages as well as activity traces of a control dam (yellow) and ELS dam (pink) over a 24-h period with her pups, traced in EthoVision. **e** Plots of the mean weight for control (closed) and stressed (open) male (blue) and female (red) mice across development. For weight plot, data represent means ± 1 SEM. Main effects depicted as #; #*p* < 0.001

Recent work in rodents have identified sex differences in neurobiological vulnerability to ELS [31–33]. However, few animal models reliably model sex differences in behavioral phenotypes of stress-associated affective pathology [15, 34–36], and fewer find a specific risk in females [37]. Here we tested if ELS in the form of fragmented maternal care, induced by limited bedding (from P4 to P11), resulted in sex differences in the development of depressive- or anxiety-like behaviors. In tandem, we used newly developed computer vision behavioral recognition tools to assess symptom development as well as their responsiveness to ketamine, a new antidepressant treatment. ELS effects on affective behavioral outcomes were tested in both male and female mice during early adolescence (P34–P38) or adulthood (P65–P75) and on home cage behavior during the young adult period (P55–P60) (Fig. 1a–c). Using both traditional tests and computer vision tracking techniques, we established that in young adulthood, only females went on to develop depressive-like behaviors, and that these symptoms could be rescued with the fast-acting antidepressant ketamine.

## Materials and methods

### Subjects

Male and female C57BL/6N mice were bred in house, had ad libitum access to food and water, and were housed on a 12 h:12 h light cycle. All animal procedures were approved by the Brown University Institutional Animal Care and Use Committee and consistent with the guide for the care and use of animals in research. With a few exceptions (see supplemental methods) independent naive groups of animals were tested in each behavioral assay to limit stress associated with handling and testing (see Fig. 1a–c for timeline).

### Limited bedding induced fragmentation of maternal care

Limited bedding paradigm was as described by Bath et al. [16]. Briefly, 4 days following birth of a litter, the dam and pups were transferred from their standard home cage to a home cage with a wire mesh bottom and a 2 × 4 cm cotton nestlet as their only source of bedding and remained in these conditions for 1 week (Fig. 1d) [16, 23]. Control mice were left undisturbed throughout these procedures. All pups were weaned and sex segregated at 21 days of age. Previous work in mice has shown that bedding restriction leads to fragmented maternal care, a chaotic early environment for developing pups [16, 23, 38]. As previously reported, this manipulation led to significant effects on nest departures of the dam (Fig. 1d) and somatic development in both male and female pups (Fig. 1e). ELS animals weighed an average of 17.4% less than control animals across development (*F*_(19,380)_ = 167.048, *p* < 0.000) [16, 39]. All animals gained weight across development *F*_(19,380)_ = 2467.723, *p* < 0.000), with males weighing more than females *F*_(19,380)_ = 101.357, *p* < 0.000), and no significant three-way interaction between sex × rearing condition × age, suggesting the effects of ELS on weight gain were similar across sexes.

### Sucrose preference test

This test was used to assess anhedonia-like behavior and is described in detail in the supplemental methods.

### Forced swim test

This test was used to assess depressive-like behavior, or despair, as previously described [40–42] in naive mice. See supplemental methods.

### Novelty-induced hypophagia

This task was used to quantify motivation and risk assessment in an unfamiliar environment in the presence of a rewarding stimulus, and was performed as previously described [43, 44] in naive mice. See supplemental methods.

### Elevated plus maze

Anxiety-like behavior was tested in the elevated plus maze (EPM) in accordance with previously used protocols [41, 45, 46]. See supplemental methods.

### Open field test

Anxiety-like behavior was tested in the open field test (OFT) in accordance with previously used protocols [41, 45, 46]. See supplemental methods.

### Continuous home cage video monitoring

Continuous home cage video monitoring was carried out as described by Jhuang et al. [47]. Mice were singly housed in a novel home cage and monitored for 5–11 days, depending on the experiment. Single housing may have served as an additional minor stressor in this group of mice. Time engaged in a given behavior per hour was averaged across days for each animal. For ketamine treatment, drug was delivered intraperitoneally at a dose of 0.6 mg/kg [48]. Data from the day of injection were eliminated from the analysis due to potential effects of handling, injection, or acute effects of ketamine on basal behaviors.

### Statistics

Two-way analysis of variance (ANOVA) was used to test for main effects of sex and rearing condition and three-way ANOVAs were used for tasks employing multiple days of testing. Follow-up one-way ANOVAs were performed with significant post hoc Bonferroni comparisons reported. For continuous home cage video monitoring, a general linear model repeated-measures ANOVA with 24 levels of hour and either two levels of treatment (control and ELS) and two levels of sex (male and female), or four levels of treatment (control, ELS, control post ketamine, and ELS post ketamine) were used to test for main effects of treatment and effect of time of day for each behavior. Alpha was set at 0.05.

## Results

### ELS is associated with sex-selective development of depressive-like behaviors in early adolescence and adulthood

**Fig. 2:**
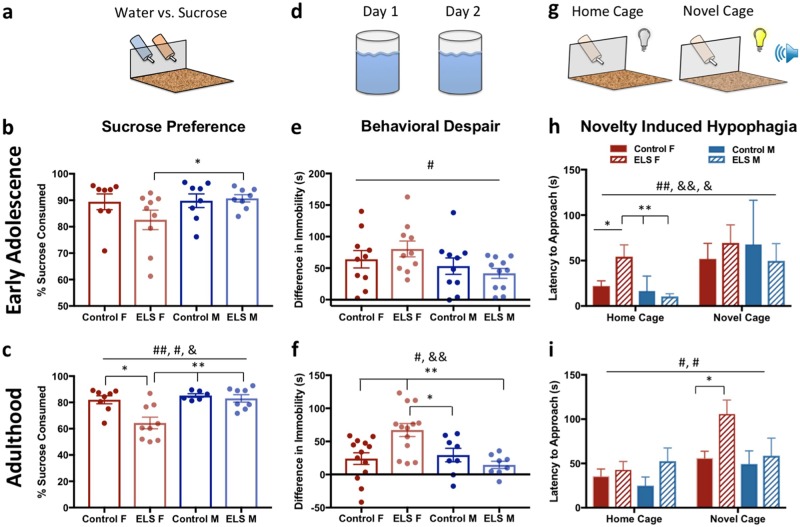
Early-onset depression in female mice exposed to ELS strengthens by adulthood. **a** For sucrose preference, animals had a choice between water or sucrose for 3 days, percent of all liquid consumed that was sucrose is reported here. **b** There was no main effect of sex or treatment at early adolescence. However, a trend toward decreased sucrose preference (anhedonia) was found in ELS females (*n* = 9) compared with ELS males (*n* = 8). **c** In adulthood, a significant main effect of sex and treatment as well as a sex × treatment interaction were found on sucrose preference, with female ELS animals (*n* = 9) driving these effects, drinking significantly less sucrose water than any other group (*n* = 7–9). In the forced swim test, **d** immobility on day 2 − immobility on day 1 (baseline) is reported here. **e** At early adolescence, an overall effect of sex was found, with females showing greater learned helplessness overall (*n* = 10–11). **f** At adulthood, a main effect of sex as well as a significant sex × treatment interaction was found, again with ELS females (*n* = 13) driving these effects, showing significantly greater behavioral despair compared with any other group (control F, *n* = 13; control and ELS M, *n* = 8). **g** In the novelty-induced hypophagia task, latency to approach the sweet milk is reported in the home and novel cage. **h** In early adolescence, all animals took longer to approach the sweet milk in the novel cage than their home cage. Specifically, in their home cage, ELS female mice (*n* = 9) took longer to approach the sweetened milk compared with all other groups (*n* = 8–9). **i** At adulthood, there were main effects of both treatment and day, with ELS animals taking longer to approach the sweetened milk than control animals, and all animals showed increased latency to approach the milk in the novel cage. There were no differences between groups in the home cage phase of the task. In the novel cage, a significant effect was found between groups specifically with ELS females (*n* = 12) taking longer to approach the sweetened milk than control females (*n* = 12). For all plots, data show means ± 1 SEM. Significance is denoted as follows: # for main effects, & for interaction effects, and * for significant post hoc comparisons; *p < 0.05, **p < 0.005. In the case of a significant one-way analysis of variance, *F* statistics are reported but only the significant post hoc Bonferroni comparisons are shown

To test if ELS contributes to the development depressive-like behaviors, mice were tested on the sucrose preference test, forced swimming test, and novelty-induced hypophagia (NIH) test. For sucrose preference (Fig. 2a), no main effect of sex or rearing condition was found at early adolescence for percent sucrose solution consumed (Fig. 2b). However, a trend toward decreased sucrose preference (anhedonia) was found in ELS females compared with ELS males (*t*(18) = 2.176, *p* = 0.050). In adulthood, a significant main effects of sex (*F*_(3, 26)_ = 9.720, *p* = 0.004) and rearing condition (*F*_(3,26)_ = 7.371, *p* = 0.012) were found, as well as a sex × rearing condition interaction (*F*_(3,26)_ = 4.433, *p* *=* 0.045) on sucrose preference (Fig. 2c). ELS females drank significantly less sucrose solution (*F*_(3,26)_ = 8.121, *p* = 0.001) than control females (*p* = 0.009), control males (*p* = 0.003), and ELS males (*p* = 0.002).

In the forced swim test, behavioral despair was calculated as the change in immobility from day 1 to day 2 (Fig. 2d). At early adolescence, a two-way ANOVA revealed a main effect of sex, with females showing a greater degree of learned helplessness (*F*_(1,39)_ = 4.659, *p* = 0.037) (Fig. 2e). Additionally, when we break this out by day, we see a main effect of rearing environment, with both male and female ELS animals showing greater immobility than control animals (Supplementary Fig. 1), particularly on the second day of testing. In adulthood, a main effect of sex (*F*_(3,38)_ = 6.049, *p* = 0.019) as well as a significant sex × rearing condition interaction (*F*_(3,38)_ = 9.064, *p* = 0.005) was found, with females and ELS-exposed mice showing increased behavioral despair (Fig. 2f). ELS females drove these effects, displaying significantly higher levels of behavioral despair (*F*_(3,38)_ = 6.732, *p* = 0.001), compared with control females (*p* = 0.005), control males (*p* = 0.05), and ELS males (*p* = 0.003). In both early adolescent and adult animals, no difference in immobility was observed on day 1 between groups, suggesting the effects of ELS and sex on learned helplessness were not due to baseline differences in motor activity (Supplementary Fig. 1).

For the NIH test, at early adolescence, a three-way ANOVA revealed a main effect of day (*F*_(7,58)_ = 10.325, *p* = 0.002), with all animals taking longer to approach the sweet milk in the novel cage; as well as a sex × rearing condition interaction (*F*_(7,58)_ = 12.509, *p* = 0.001) and a sex × day interaction (*F*_(7,58)_ = 4.084, *p* = 0.048) (Fig. 2h). ELS female mice displayed an increased latency to approach the sweetened milk in their home cage (*F*_(3,33)_ = 6.759, *p* = 0.001) compared with control females (*p* = 0.045), control males (*p* = 0.004), and ELS males (*p* = 0.002), indicating an increase in un-provoked depressive-like behavior. In the novel cage, ELS females behaved similarly to control mice and ELS males.

In adulthood, there was a main effect of both rearing condition (*F*_(7,47)_ = 7.656, *p* = 0.007) and day (*F*_(7,74)_ = 6.588, *p* = 0.012), with ELS animals taking longer to approach the sweetened milk than control animals, and all animals showing increased latency to approach the milk in the novel cage (Fig. 2i). No differences were found between groups in the home cage task phase. In the novel cage, a significant effect of group was found (*F*_(3, 37)_ = 3.147, *p* = 0.036), specifically with ELS females taking longer to approach the sweetened milk than control females (*p* = 0.047). No effect was found on motivational parameters (Supplementary Fig. 2a, b)

### No sex-selective anxiety-like phenotypes were observed in ELS-exposed mice during early adolescence or adulthood in traditional behavioral tests

**Fig. 3:**
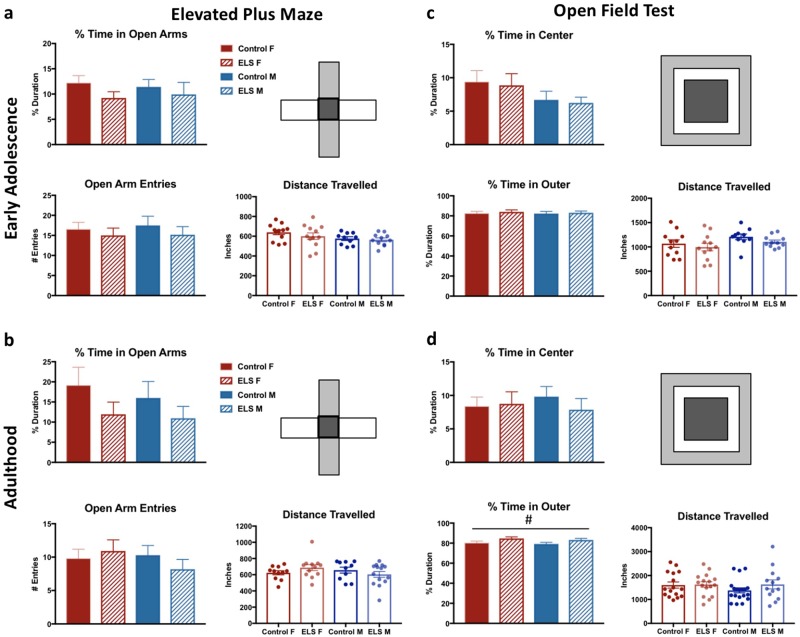
Early life stress does not lead to sex-selective anxiety-like behavior in early adolescence or adulthood. **a**, **b** The elevated plus maze measures anxiety as a % duration of time spent exploring the open arms (white) and number of entries into the open arms. At early adolescence (*n* = 10–12) and adulthood (*n* = 10–12), no differences were detected between groups on either measure. Additionally, there were no effects of sex or rearing condition on locomotor activity in this task. **c**, **d** The open field arena is divided into three zones: outer (light gray); middle (white); and center (dark gray). Anxiety is measured by decreased % time spent in center and increased % time spent in outer areas. **c** No effects of sex or rearing condition were observed on anxiety or locomotion in early adolescent mice (*n* = 10–12). **d** In adult mice, there was a significant effect of rearing condition on % time in outer arena, with ELS spending more time in this area, but no effects of sex or rearing condition on % time in center or locomotion were observed (*n* = 16). For all plots, data represent means ± 1 SEM. Main effects depicted as #; #*p* < 0.05

During early adolescence and adulthood, no main effects of sex or rearing condition were detected between groups on either the OFT or the EPM, measures of anxiety-like behavior. Additionally, there were no effects of sex or rearing condition on locomotor activity, as measured by distance traveled during the task (Fig. 3a, b).

In the open field test, no effects of sex or rearing condition were observed on anxiety-like behavior or locomotion in early adolescent mice (Fig. 3c). In adult mice (Fig. 3d), there were no effects of sex or rearing condition observed on percent time in center or distance traveled. However, there was a significant effect of rearing condition on percent time in outer arena, with all ELS animals spending more time in this area than control animals (*F*_(3,62)_ = 5.134, *p* = 0.027). No effect of sex or sex × rearing condition interaction was observed.

### Continuous home cage video monitoring in early adulthood reveals otherwise undetectable depressive-like behaviors in female mice exposed to ELS

We used our newly developed method of continuous video monitoring of home cage behavior of mice [47] to test for depressive-like outcomes following ELS. We focused on a suite of behaviors that are analogous to the broader behavioral profile of depression in humans [49], including sleep disturbance (resting), lethargy (walking), and diminished self-care (grooming), and continuously tracked these behaviors over several days (5–7 days). Behavior for each animal was averaged across 5 days of recording into a single value for each hour of the 24-h circadian period and then averaged within group.

### Grooming

**Fig. 4:**
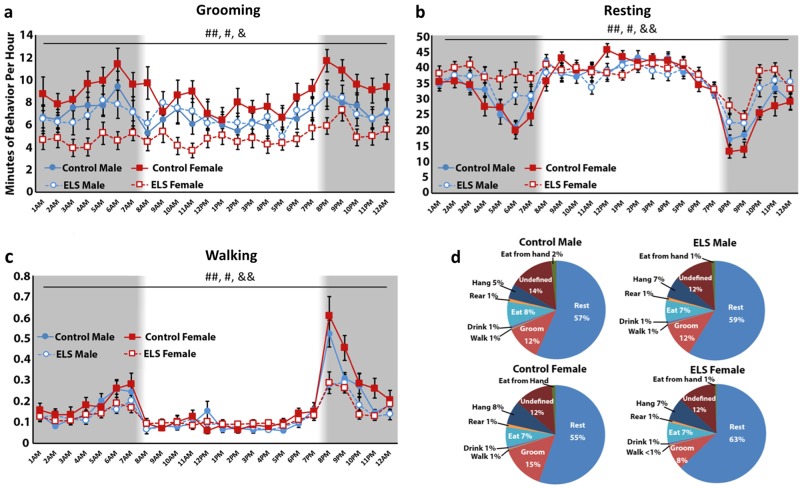
Early life stress leads to depressive-like home cage behaviors, specifically in female mice. Ethograms presenting the mean time (minutes ± 1 SEM) engaged in a given behavior for each hour over a 24-h period, averaged over 5 days. Mice were housed on a 12:12 light:dark cycle, with shaded regions indicating periods of dark and white regions indicating periods of light. Measures are presented for male control (*n* = 15, closed circles) and ELS (*n* = 12, open circles) mice, shown in blue, as well as female control (*n* = 13, closed squares) and ELS (*n* = 18, open squares) mice, in red. **a** ELS females show diminished self-care, spending significantly less time grooming than control females and male animals at hours 3 (*F*_(3,57)_ = 4.860, *p* = 0.005), 4 (*F*_(3,57)_ = 5.151, *p* = 0.003), 5 (*F*_(3,57)_ = 3.315, *p* = 0.027), 6 (*F*_(3,57)_ = 6.347, *p* = 0.001), 7 (*F*_(3,57)_ = 3.386, *p* = 0.025), 8 (F_(3,57)_ = 4.747, *p* = 0.005), 10 (*F*_(3,57)_ = 3.042, *p* = 0.037), 11 (*F*_(3,57)_ = 6.581, *p* = 0.001), 18 (*F*_(3,57)_ = 3.234, *p* = 0.029, 20 (*F*_(3,57)_ = 6.033, *p* = 0.001), 22 (*F*_(3,57)_ = 4.473, *p* = 0.007), and 23 (*F*_(3,57)_ = 3.447, *p* = 0.023). This stress-effect was sex-selective, with ELS males performing similar grooming patterns to control males. **b** ELS females spent significantly more time sleeping than control females and male animals. Specifically, significant effects of group were found during hours 5 (*F*_(3,57)_ = 3.288, *p* = 0.027), 6 (*F*_(3,57)_ = 10.235, *p* < = 0.001), 7 (*F*_(3,57)_ = 3.454, *p* < 0.023), 12 (*F*_(3,57)_ = 3.272, *p* = 0.028), 20 (*F*_(3,57)_ = 9.135, *p* < 0.001), 21 (*F*_(3,57)_ = 4.597, *p* = 0.006), 22 (*F*_(3,57)_ = 10.526, *p* < 0.001), and 23 (*F*_(3,57)_ = 3.847, *p* < 0.014), which are almost all during the animals’ dark cycle. **c** Exclusively during dark hours, ELS females demonstrated reduced locomotion, spending significantly less time walking than control animals. Significant effects of group were found during hours 20 (*F*_(3,57)_ = 6.947, *p* < 0.001), 21 (*F*_(3,57)_ = 4.794, *p* = 0.005), 22 (*F*_(3,57)_ = 3.165, *p* = 0.032), and 23 (*F*_(3,57)_ = 5.994, *p* = 0.001). **d** Detailed breakdown of the percent of time that each group of animals engaged in specific behaviors over the course of 5 days in their home cage

For grooming, a significant effect was found for hour (*F*_(23,1242)_ = 6.142, *p* < 0.001), with variation in the level of grooming across the circadian cycle (Fig. 4a). There was no main effect of sex (*F*_(1,54)_ = 0.023, *p* = 0.880), but a significant effect of rearing condition (*F*_(1,54)_ = 6.812, *p* = 0.012) as well as a sex × rearing condition interaction (*F*_(1,54)_ = 6.573, *p* = 0.013) was observed. In follow-up comparisons, ELS led to decreased grooming during 12 distinct hours (Fig. 4a), mostly during the dark portion of the animals’ circadian cycle. In independent analyses of male and female behavior, females showed a significant effect of rearing condition on grooming behavior (*F*_(1,29)_ = 16.825, *p* < 0.001), with a significant decrease in grooming in ELS compared with control reared mice. For males, no significant effect of rearing condition was found for grooming behavior (*F*_(1,25)_ = 0.001, *p* = 0.977).

### Resting

For resting, a significant effect was found for hour (*F*_(23,1242)_ = 30.507, *p* = 0.001) with variation in levels of resting over the circadian cycle (Fig. 4b). A significant hour × condition interaction was also observed (*F*_(23,1242)_ = 5.052, *p* < 0.001) with ELS animals engaging in greater resting behavior than control reared mice. While no significance between-subjects effect of sex was observed (*F*_(1,54)_ = 0.820, *p* = 0.369), a significant main effect of rearing condition (*F*_(1,54)_ = 9.213, *p* = 0.004) and a marginal sex × rearing condition interaction (*F*_(1,54)_ = 2.541, *p* = 0.117) were found. Specifically, for females, there was a significant effect of rearing condition (*F*_(1,29)_ = 9.734, *p* = 0.004), with ELS females engaging in greater resting behavior than control reared females (Fig. 4b). For males, no significant effect of rearing condition was found for resting behavior (*F*_(1,29)_ = 1.225, *p* = 0.279). In ELS females, the significant elevation in resting behavior was most apparent during the period preceding lights on in the housing room (~hour 7) and following lights off in the housing room (~hour 19) (Fig. 4b).

### Walking

For walking, a significant effect of hour was observed (*F*_(23,1242)_ = 43.268, *p* < 0.001), with changes in walking over the circadian cycle, as well as an hour × rearing condition interaction (*F*_(23,1242)_ = 6.216, *p* < 0.001) (Fig. 4c). A significant main effect of rearing condition (*F*_(1,54)_ = 7.255, *p* = 0.009) was also found, but no main effect of sex (*F*_(1,54)_ = 1.324, *p* = 0.255) or sex × condition interaction (*F*_(1,54)_ = 0.829, *p* = 0.367). For female mice, a significant effect of rearing condition was found for walking behavior (*F*_(1,29)_ = 5.031, *p* = 0.033), with ELS females engaging in less walking behavior than control reared animals (Fig. 4c). No effect of rearing condition was found for walking behavior in males (*F*_(1,25)_ = 2.655, *p* = 0.116).

A detailed characterization of the effects of ELS on additional home cage behaviors in male and female mice can be found in Supplementary Information. Here we show a breakdown of the distribution of behaviors for control and ELS-exposed male and female mice over the course of 5 days of recording (Fig. 4d). Additional behaviors are analyzed by hour, and differences between groups are reported for drinking, eating, eating from hand, rearing, and hanging behaviors, as well as undefined movements (e.g. movements that could not be categorized into one of the other behavioral classes) (Supplementary Fig. 3).

### Ketamine rapidly rescues home cage depressive phenotype in female mice

**Fig. 5:**
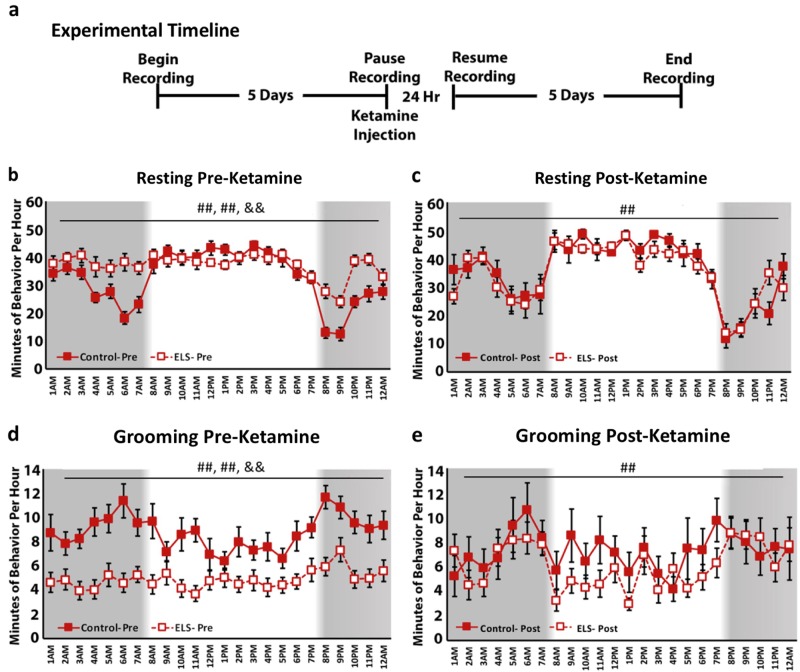
The fast-acting, atypical antidepressant, ketamine, rescues depressive-like home cage behavior in ELS female mice. **a** Experimental design. **b** Significant differences in resting behaviors exist between control and ELS females before ketamine treatment, specifically at hour 4 (*t*_29_ = 2.516, *p* = 0.018); hour 6 (*t*_29_ = 4.710, *p* < 0.001); hour 7 (*t*_29_ = 3.579, *p* = 0.001); hour 12 (*t*_29_ = 2.091, *p* = 0.045); hour 13 (*t*_29_ = 2.248, *p* = 0.032); hour 20 (*t*_29_ = 4.351, *p* < 0.001); hour 21 (*t*_29_ = 3.612, *p* = 0.001); hour 22 (*t*_29_ = 4.942, *p* < 0.001); and hour 23 (*t*_29_ = 3.585, *p* < 0.001). **c** Ketamine treatment reverses disordered resting behavior in ELS females to match control animals. There only remaining effect of rearing condition exists at hour 23 (*t*_29_ = 2.160, *p* = 0.046). **d** Prior to ketamine treatment, ELS females spend significantly less time grooming than control females at nearly every hour (hour 1 (*t*_29_ = 2/588, *p* = 0.015); hour 2 (*t*_29_ = 2.285, *p* = 0.030); hour 3 (*t*_29_ = 3.793, *p* = 0.001); hour 4 (*t*_29_ = 4.094, *p* < 0.001); hour 5 (*t*_29_ = 3.069, *p* = 0.005); hour 6 (*t*_29_ = 4.489, *p* < 0.001); hour 7 (*t*_29_ = 3.645, *p* = 0.001); hour 8 (*t*_29_ = 3.385, *p* = 0.002); hour 10 (*t*_29_ = 3.063, *p* = 0.005); hour 11 (*t*_29_ = 4.717, *p* < 0.001); hour 14 (*t*_29_ = 2.693, *p* = 0.12); hour 15 (*t*_29_ = 2.053, *p* = 0.049); hour 16 (*t*_29_ = 2.525, *p* = 0.017); hour 17 (*t*_29_ = 2.151, *p* = 0.040); hour 18 (*t*_29_ = 2.906, *p* = 0.007); hour 19 (*t*_29_ = 2.590, *p* = 0.015); hour 20 (*t*_29_ = 4.797, *p* < 0.001); hour 21 (*t*_29_ = 2.505, *p* = 0.018); hour 22 (*t*_29_ = 4.074, *p* = < 0.001); hour 23 (*t*_29_ = 3.127, *p* = 0.004); and hour 24 (*t*_29_ = 2.648, *p* = 0.013)). **e** Following ketamine treatment, ELS females engaged in self-care (grooming) to the same extent as control females. No differences exist between groups at any hour. For all plots, data represent mean ± SEM. Significance is denoted as follows: # for main effects and & for interaction effects, ^#^*p* *<* 0.05, ^##^*p* *<* 0.001

To test whether a single dose of ketamine could rescue the effect of ELS on home cage depressive-like behavior, we focused on two metrics, grooming (decreased self-care) and resting (circadian disruption), in a new cohort of mice. Control and ELS female mice were recorded for 5 days to obtain baseline behavior (pretreatment), after which all animals received a single intraperitoneal injection of 0.6 mg/kg of ketamine. The day following injection, recording resumed for an additional 5-day period (post treatment) (Fig. 5a).

### Ketamine rescues elevated resting phenotype in ELS reared females

For resting, prior to ketamine treatment, a significant main effect was found for hour (*F*_(23,667)_ = 16.455, *p* < 0.001) as well as an hour × rearing condition interaction (*F*_(23,667)_ = 5.073, *p* < 0.001). A significant between-subjects effect of rearing condition was also observed (*F*_(1,29)_ = 12.344, *p* = 0.001), with ELS female mice engaging in significantly more resting behavior than control reared mice (Fig. 5b). These results replicated the initial observation that ELS female mice show a flattened circadian cycle and engage in greater resting behavior than control.

Following treatment, there continued to be a main effect of hour (*F*_(23,368)_ = 18.594, *p* < 0.001) on resting behavior. However, during the post-treatment period the hour × rearing condition effect was no longer significant (*F*_(23,368)_ = 1.060, *p* = 0.388). In addition, the between-subjects effect of rearing condition was no longer significant (*F*_(1,16)_ = 0.243, *p* = 0.628) with ketamine-treated ELS mice now resembling the behavior of controls (Fig. 5c). The only hour in which groups significantly differed in the level of resting behavior following ketamine treatment was hour 23 (*p* = 0.046).

### Ketamine rescued reduced grooming phenotype in ELS female mice

For grooming, prior to ketamine treatment, a significant main effect of hour (*F*_(23, 667)_ = 3.833, *p* < 0.001) as well as an hour × rearing condition interaction (*F*_(23,667)_ = 1.979, *p* = 0.004) was observed. When investigating between-subjects effects, a significant effect of rearing condition was found (*F*_(1,29)_ = 16.825, *p* < 0.001), with ELS female mice engaging in significantly less grooming behavior than control reared mice (Fig. 5d), replicating our previous results.

Following ketamine treatment, the significant effect of hour persisted (*F*_(23,368)_ = 3.192, *p* < 0.001). However, there was no longer an hour × treatment interaction (*F*_(23,368)_ = 1.126, *p* = 0.314) or a significant main effect of group (*F*_(1,16)_ = 0.784, *p* = 0.389) (Fig. 5e). Ketamine treatment normalized levels of grooming behavior in ELS mice to control levels.

Together, these data provide further support that ELS in the form of fragmented maternal care causes depressive-like outcomes selectively in female mice. We have shown that these sex-selective, stress-induced behaviors are rescued with the atypical antidepressant ketamine. Here we have evidence that home cage monitoring is an effective, noninvasive method for assessing these depressive-like behaviors, studying the potential mechanisms underlying stress-induced pathology, and for testing potential pharmacological interventions.

## Discussion

Here ELS in the form of limited bedding led to a developmentally emergent depressive-like phenotype that was present during early adolescence and became more robust in adulthood. ELS females showed symptoms of anhedonia; behavioral despair; as well as decreased motivation to approach a sweet reward in an anxiogenic environment (only in adulthood). While many of these effects were equally present in adolescent and adult mice, one noted difference was observed in the NIH task. Adolescent ELS females demonstrated a more innate anhedonic response to the sweet reward in their home cage, taking longer to approach than other groups; while in the novel cage environment, there were no observed effects of ELS or sex. In general, adolescent mice appeared to be more affected by the novel environment, with increased latencies and decreased time spent drinking in the new cage in comparison with adult mice (Supplementary Fig. 2). Alternatively, adult ELS females showed decreased motivation to approach the reward only in the novel cage, indicating that under home cage conditions, the reward was salient enough to similarly motivate these animals. Interestingly, depressive-like behaviors in ELS-exposed females were not comorbid with anxiety-like phenotypes. As measured in the EPM and OFT, ELS did not lead to anxiety-like behavior in either sex in early adolescence and caused only a slight decrease in exploratory behavior in adult animals, in both sexes. Effects seen in the depressive- and anxiety-like behavior tasks could not be explained by effects of ELS on locomotor activity. Further, using continuous home cage video monitoring, we detected additional, discrete depressive-like behaviors in ELS-exposed females. Specifically, we observed diminished self-care (grooming), decreased walking, and increased resting over the circadian cycle. Behavioral disturbances in ELS females mimicked core symptoms of depression in humans [50], and were rescued by the fast-acting antidepressant, ketamine. While we do anticipate some potential sex-selective shifts in the onset of puberty as a result of this ELS manipulation (Manzano-Nieves, in review), we did not directly assess effects of estrous cycle stage on anxiety or depressive-like behavior in this manuscript [41]. However, to account for such effects, a large number of animals from multiple litters were sampled to increase the probability of representation from multiple cycle stages in a given task. Additionally, adolescent animals were tested before the onset of estrous, and sex-selective effects of ELS were already observed in these mice. It is also possible that single housing associated with automated continuous monitoring served as a secondary mild stressor, magnifying the depressive-like phenotype in ELS female mice in home cage recording experiments. However, the presence of depressive-like behaviors in home cage monitoring, as well as across traditional behavioral testing, which were carried out in independent cohorts of group housed mice, indicates that the depressive-like phenotype in ELS females can be observed regardless of housing conditions.

Clinical and experimental studies have shown that ELS is associated with the later development of stress-associated pathology, including major depressive disorder (MDD) and anxiety [1, 3, 51–53]. In fact, a single, significant stressor during childhood can increase the risk for pathology by ~30% [1]. In many cases, stress involves disruption of the mother–child relationship (e.g. neglect, maternal depression, parental loss, maternal psychopathology, or abuse) [27, 50, 52, 54]. The developmental nature of the stressor can greatly influence the timing of pathology emergence. Disruptions during sensitive, early developmental stages, may alter developmental trajectories leading to emergent pathological outcomes with symptoms surfacing in adolescence or young adulthood [55–57]. In humans, sex is a reliable risk factor for depression and anxiety, with females being nearly two times more likely than males to be affected [55, 58, 59]. In a review, etiology and lifetime comorbidities between MDD and generalized anxiety disorder were assessed [60] finding that these disorders are often co-diagnosed [60, 61]. However, there is evidence that different types of early stressful events are more likely to result in one disorder over the other [4, 53, 62–64]. For instance, parental separation is associated with both disorders equally, but reduced parental care and childhood abuse were more prevalent in patients with MDD [60, 65]. The nature of the fragmented maternal care model used here may more accurately model childhood neglect and abuse, leading to the depressive but not anxiety-like phenotype in ELS-exposed females.

Despite the heightened female risk for both anxiety and depression, many ELS paradigms in rodents fail to replicate these sex effects [66]. This could be due to insensitivity of some traditional, short-term behavioral measures that are susceptible to differences in developmental stage and estrous cycle. To diagnose depression, a person must report (1) depressed mood or a loss of interest in daily activities for more than 2 weeks and (2) five of the nine core symptoms of MDD, nearly every day [67]. Yet, despite the complexity of MDD, measures of depressive-like behavior in animal models are acute measures (such as forced swim or tail suspension), while continuous measures such as changes in sleep and motor activity, self-care, motivation, or anxiety are often overlooked.

Here we have addressed the need for more robust methods to study depressive-like behaviors in mice, which are more relatable to the complex constellation of symptoms observed in human pathology. We supplemented traditional depressive- and anxiety-like behavior assays with the use of novel, noninvasive automated home cage continuous video monitoring. Using this system, we were able to detect robust behavioral markers of affective pathology over long spans of time and without the stress associated with typical behavioral methodologies. Using this combined approach (traditional and novel home cage assessment) we uncovered a female-selective depressive-like phenotype following ELS that emerged by early adolescence and strengthened in adulthood. Collectively, the data presented here provides a comprehensive picture of the effect of ELS on behavioral and affective outcomes in both male and female mice. The sex difference in risk for pathology opens up the possibility to leverage this model to understand sex differences in risk as well as possible factors mediating risk and resilience for stress-associated pathology.

Using this system, we find that ketamine has fast-acting antidepressant effects in ELS-exposed females; reversing home cage depressive-like behaviors, mirroring the rapid alleviation of core major depression symptoms following an intravenous dose of ketamine in otherwise treatment-resistant patients [68, 69]. The current findings are even more powerful as the video monitoring method allowed for a within-subject design, assessing the same metrics pre- and post treatment in the same animal. Use of traditional behavioral methods (forced swim, tail suspension, etc.), can only be employed a single time in a given animal, resulting in the need for either a cross-sectional approach or the use of different assays to assess outcomes from what were used prior to treatment. The novel home cage monitoring technique described here has the potential to be useful for depression screening in rodent models and long-term efficacy and safety testing of new pharmacological interventions, to identify better interventions for treatment-resistant MDD patients. By rescuing ELS-induced behavioral effects with a known antidepressant, we have revealed construct validity of this testing method for depression and drug screening. Quantifying home cage behaviors such as walking, sleeping, eating, and grooming over long periods of time could provide a significant benefit over the current gold standards of pharmacological testing. In addition to being one-dimensional, traditional behavioral tests (forced swim, tail suspension, etc.) have been shown to be sensitive to such external factors as the gender of the experimenter [70]. Continuous home cage monitoring eliminates effects of animal handling, time of day, temperature, estrous cycle, or user biases on testing outcomes, providing a more clean and thorough analysis.

Further research into the mechanism driving these sex-selective, ELS-induced depressive-like behaviors, is necessary. In previous work, we find that fragmented maternal care causes accelerated maturation of hippocampus by truncating neurodevelopment [16]. In males, ELS led to an earlier developmental silencing of cell proliferation and acceleration of parvalbumin interneuron cell maturation in the hippocampus and an accelerated development of contextual fear learning [16]. More work in females to assess ELS effects on the timing of regional brain maturation is necessary, as sex-selective disruption in neurodevelopmental timing may contribute to sex-selective development of depressive-like behaviors in female mice. Supporting this notion, we have found that ELS leads to sex disparities in cognitive outcomes, impairing spatial memory in male mice but sparing this behavior in females [39]. The current work lays the foundation for further exploration of the possible neurobiological underpinnings of these sex-selective effects on affective development, and mechanisms that underlie female risk for stress-associated mental illness.

In summary, this is the first time traditional depressive- and anxiety-like behavioral assays have been used in combination with novel home cage continuous video tracking methods to report a female-specific depressive-like phenotype following ELS. Fragmented maternal care led to a sex difference in risk for pathology that mirrors behavioral outcomes in the human population. This sexual dimorphism will allow for future in-depth analyses of female-specific vulnerability and male-specific resilience to affective pathologies following ELS. Additionally, we have demonstrated that depressive-like behaviors in ELS female mice can be rescued with a fast-acting, atypical antidepressant, suggesting the observed behavioral changes are analogous to human symptoms of depression, and offering a basis for understanding the molecular underpinnings of stress-induced disruption. Further, we have provided evidence that continuous home cage monitoring is an advantageous novel tool for depression and drug screening in rodent models.

## Electronic supplementary material

Supplemental Material
