# Supplementary material for: Early life stress leads to sex differences in development of depressive-like outcomes in a mouse model
Source: Neuropsychopharmacology. 2018 Sep 6;44(4):711–20. doi: 10.1038/s41386-018-0195-5 (PMC6372611; doi:10.1038/s41386-018-0195-5)
Supplement: Supplementary file 1 — Supplemental Material [file 41386_2018_195_MOESM1_ESM.pdf]

SUPPLEMENTAL INFORMATION

This Supplemental Information includes

-Supplemental Data

Figure S1, related to Figure 2
Figure S2, related to Figure 2
Figure S3, related to Figure 4

-Supplemental Methods

-Supplemental Results

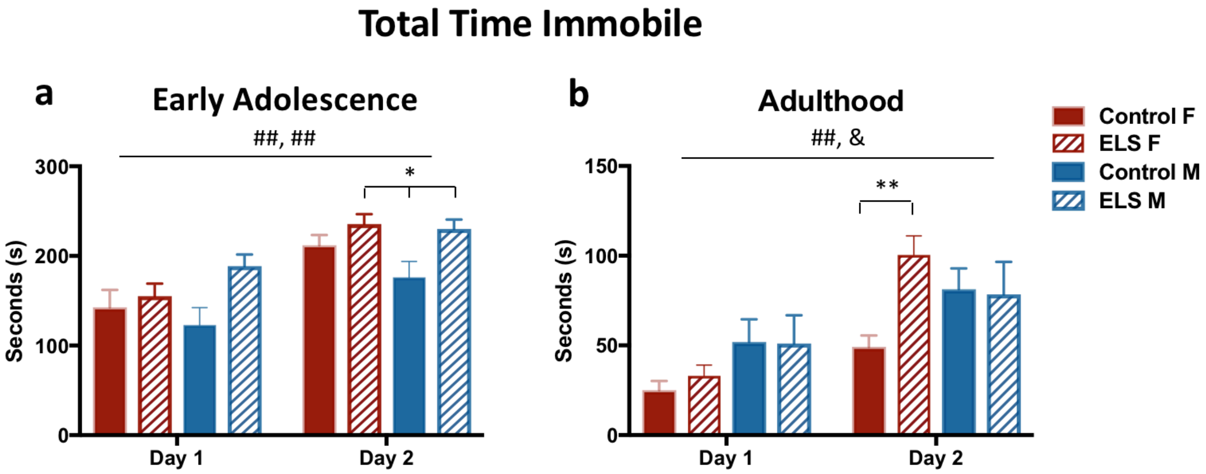

Supplemental Figure 1. *Sex-selective ELS-induced learned helplessness is not due to differences in mobility in the forced swim test.* (a) In early adolescence, all groups spend more time immobile on day 2 in comparison to day 1 (main effects of day ($F_{(7,74)}$ = 32.063, p < 0.000)), with ELS animals displaying significantly more learned helplessness ness than control animals (main effect of treatment ($F_{(7,74)}$ = 12.994, p = 0.001)). Importantly, there are no differences on Day 1 between groups, suggesting the behavioral despair is not due to differences in mobility. on Day 2, there is a significant difference between groups $F_{(3,37)}$ = 4.131, p = 0.013, with female and male ELS animals spending more time passively floating than control males (p = 0.019 and p = 0.033, respectively). (b) In adult animals, all groups show an increase in immobility on Day 2 (main effect of Day ($F_{(7,78)}$ = 23.966, p <0.000)). There is also a significant sex x treatment interaction ($F_{(7,78)}$ = 4.317, p = 0.41). Again, we observe no differences between groups on Day 1, suggesting no baseline differences in mobility in the water. On Day 2, there is a significant difference between groups ($F_{(3,39)}$ =4.474, p = 0.009) , specifically with ELS females spending more time passively floating than control females, (p = 0.005). For all plots, error depicts standard error of the mean. Significance is denoted as follows: # for main effects, & for interaction effects, * for significant post-hoc comparisons; p<.05 = *, p<.005 = **. In the case of a significant one-way analysis of variance, F statistics are reported but only the significant post-hoc Bonferroni comparisons are shown.

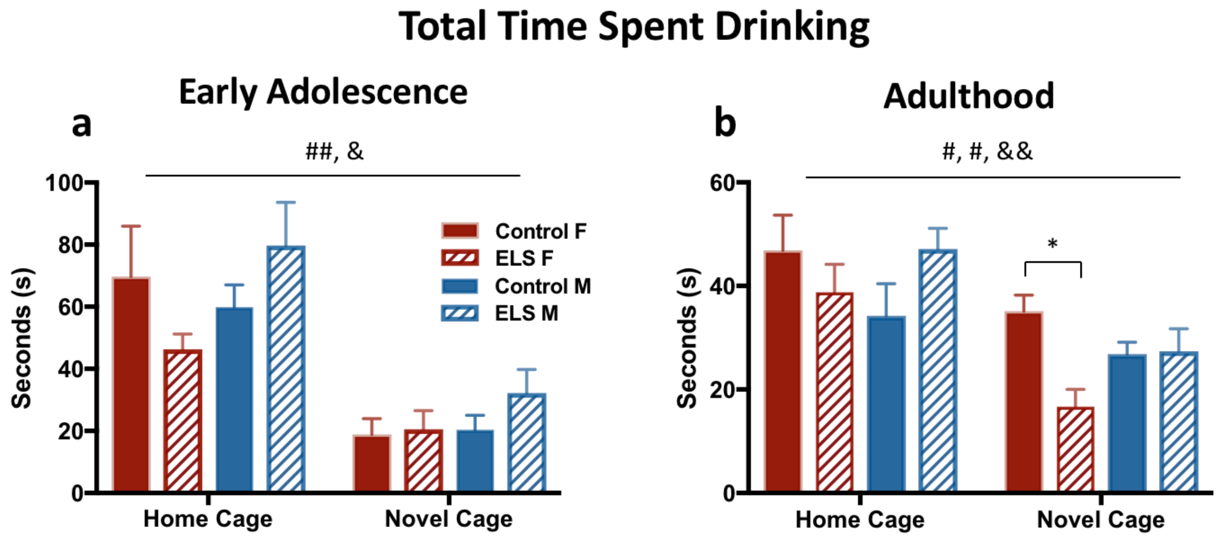

Supplemental Figure 2. *ELS leads to sex-selective depressive behavior in novelty induced hypophagia task.* Specific statistics are reported in text. (a) In early adolescence, there was a main effect of day on duration of time drinking the sweetened condensed milk with all animals drinking for less cumulative time in the novel cage. There was also a significant sex x rearing condition interaction. (b) In adult mice, there were significant main effects of day and rearing condition as well as a sex x rearing condition interaction. Differences between groups were driven by ELS females drinking for significantly less time than control females in the novel cage.

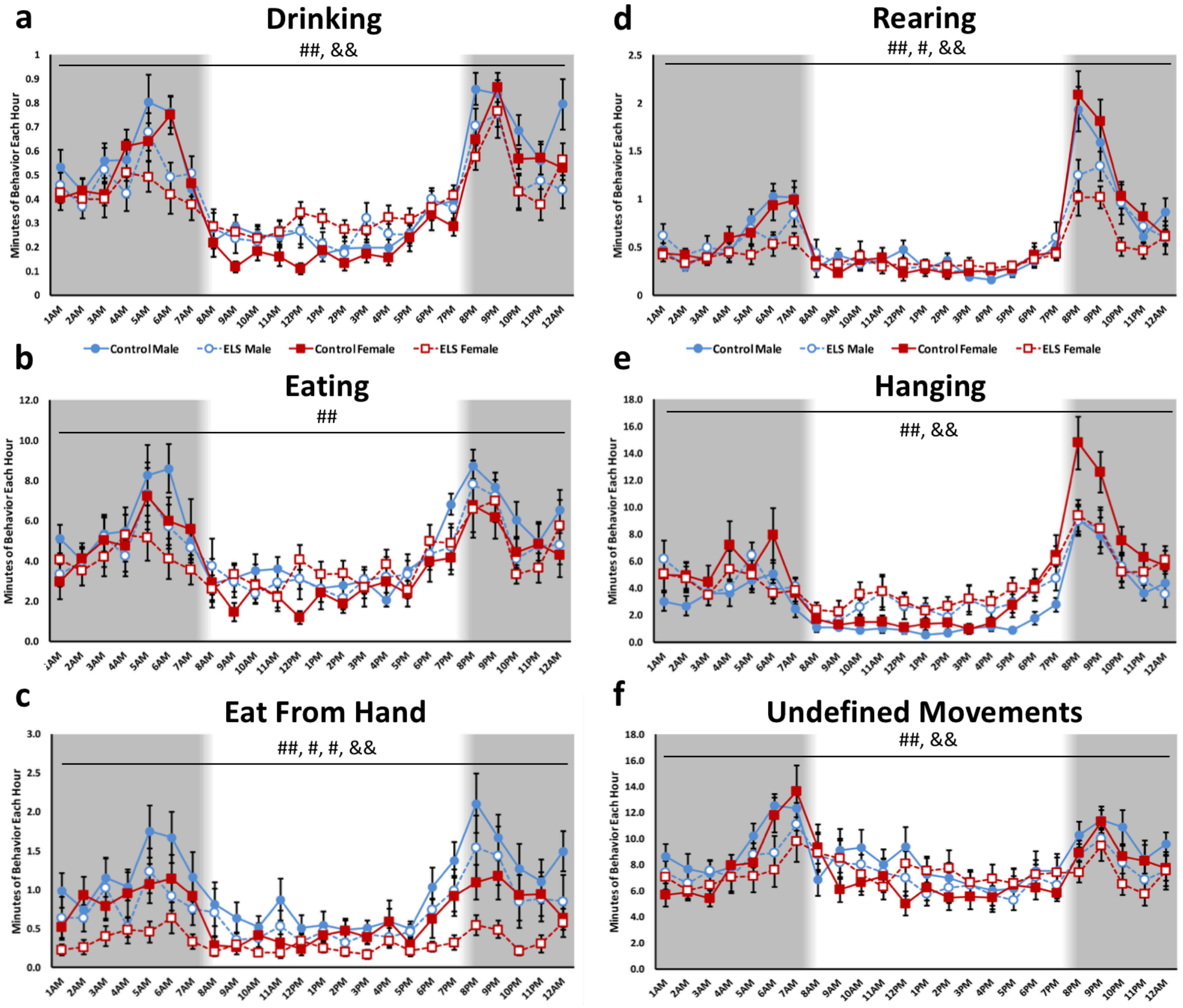

Supplemental Figure 3. *Effects of rearing condition and sex on home-cage behaviors*. Ethograms presenting the mean time (minutes ± 1 SEM) mice engaged in a given behavior for each hour over a 24-hour period, averaged over five days. Grey shaded regions indicate hours in the dark while white regions indicate periods of light. (a) Animals engaged in drinking behavior at differing frequencies throughout their dark-light cycle, which was significantly affected by rearing condition. Specifically, we observed a significant effect of group during hours 6 ($F_{(3,57)}$ = 6.054, p = 0.001), 12 ($F_{(3,57)}$ = 3.721, p = 0.017), 13 ($F_{(3,57)}$ = 2.970, p = 0.040), 16 ($F_{(3,57)}$ = 3.172, p = 0.031), 20 ($F_{(3,57)}$ = 4.141, p = 0.010), 22 ($F_{(3,57)}$ 3.665, p = 0.018), and 24 ($F_{(3,57)}$ = 3.577, p = 0.020). (b) Eating from the food hopper varied with the circadian rhythm in all animals but was not affected by sex or rearing condition. (c) Eating from hand was significantly affected by rearing condition and sex, particularly in female mice. ELS females engaged in less of this behavior during dark hours. Specifically, a significant effect of group was observed at hour 1 ($F_{(3,57)}$ = 3.365, p = 0.025), 2 ($F_{(3,57)}$ = 3.224, p = 0.030), 5 ($F_{(3,57)}$ = 5.097, p = 0.004), 6 ($F_{(3,57)}$ = 2.957, p = 0.040), 7 ($F_{(3,57)}$ = 2.934, p = 0.042) 11 ($F_{(3,57)}$ = 3.064, p = 0.036), 15 ($F_{(3,57)}$ = 2.958, p = 0.040), 18 ($F_{(3,57)}$ = 3.307, p = 0.027), 19 ($F_{(3,57)}$ = 5.093, 0.004), 20 ($F_{(3,57)}$ = 5.748, p = 0.002), 21 ($F_{(3,57)}$ = 4.165, p = 0.010), 22 ($F_{(3,57)}$ = 3.199, p = 0.030), 24 ($F_{(3,57)}$ = 3.475, p = 0.022)). (d) Rearing behavior, when a mouse stands solely on its back paws, was significantly affected by time of day, and rearing environment. Specifically, differences between groups were found at hour 6 ($F_{(3,57)}$ = 4.177, p = 0.010), 7 ($F_{(3,57)}$ =2.939, p = 0.041), 20 ($F_{(3,57)}$ = 6.075, p = 0.001), 21 ($F_{(3,57)}$ = 3.854, p = 0.014), 22 ($F_{(3,57)}$ = 3.546, p = 0.020). (e) Mice engaged in hanging behavior (climbing on the top of the

cage) differently across hours of the day. This was affected by rearing condition, with ELS animals hanging less than control animals during particular hours of the night. (f) Undefined movements could not be further characterized, but again were affected by hour and rearing condition. For all plots, data represent mean ± SEM. Significance is denoted as follows: # for main effects and & for interaction effects, $p < 0.05$ = #, $p < 0.001$ = ##.

SUPPLEMENTAL METHODS

Behavioral testing of mice on multiple procedures

Animals used in 24-hr tracking did not undergo any additional behavioral testing. At both early adolescence and adulthood, naïve animals were tested in the novelty induced hypophagia (NIH) task as well as the forced swim test (FST). Animals received no additional behavioral testing after the FST. Animals that underwent sucrose preference testing had only been previously exposed to a locomotor open field test and/or the elevated plus maze. Animals tested in early adolescence were distinct from animals tested at adulthood and young adulthood

Sucrose preference test

Control and ELS animals were housed in littermate pairs in clean cages and habituated for 48-hours to the new cage and the two-bottle paradigm. One bottle contained a 1% sucrose solution and the other contained drinking water. Following habituation, mice were given a free choice between these two bottles for a 72-hour test period (**Figure 2a**). No water or food deprivation was applied. To prevent possible effects of a side-preference on drinking behavior, the position of the bottles in the cage was switched every 24-hours during the 72-hour test phase. The intake of water and 1% sucrose solution, as well as total fluid consumption, were calculated by weighing the bottles before and after access to liquids during habituation and test phases. Sucrose preference was calculated as the percentage of sucrose solution consumed relative to the total amount of liquid drunk during the 72-hour test (sucrose solution intake/total intake x 100).[44] Drinking bottles were the same as described in the NIH task (below).

Forced Swim Test

Mice were transported to the behavioral testing room and allowed to habituate to the testing environment for 20-minutes. During testing, mice were individually placed in a glass 2-L beaker with 1600 mL of room temperature water (23-25°C). Beakers were set up in isolated cubicles and positioned in front of a camera connected to a computer running EthoVision. Activity was recorded for a 5-min pre-test on day 1. Twenty-four hours later, on Day 2, a 5-minute test was performed again to measure learned helplessness (**Figure 2b**). Passive floating (immobility) was measured during the 5-minute pre-test (Day 1) and test (Day 2); a difference score was calculated and reported as a measure of behavioral despair. Water was changed between animals to remove any residual olfactory cues. Ethovision immobility scores were confirmed on a subset of videos by human observers who were blinded to the animals' rearing condition group and sex.

Novelty Induced Hypophagia

For three consecutive days of training, mice were presented with diluted (1 : 1; milk : water) sweetened condensed milk (Carnation) for 40-minutes in their home cage in testing room one. Milk was presented in 150mL non-drip water bottles (Lixit Products; Napa, CA). On Day 4, home cage testing occurred, under dim lighting. Latency from bottle presentation to first milk-licking event and cumulative time spent drinking over the 5-min trial were manually scored by blinded observers. On day 5, animals underwent the same test but in a second room in a novel, clean cage under bright lighting with music playing and no nesting material, to induce neohypophagia

(**Figure 2c**). Lighting, volume and song were controlled for across animals. Latency and duration measures were compared between Day 4 (baseline) and Day 5 (novel cage).

Elevated Plus Maze

Mice were transported to the dimly lit testing room (approximately 20 lux) and allowed to acclimate for 20-minutes. The EPM apparatus included four white, plastic arms in the shape of a cross with 2 opposing open arms (30x5 cm each) and 2 opposing closed arms (30x5x20 cm), elevated to a height of 100 cm off the floor. The computerized tracking system, Ethovision, was used to record the number of entries into and time spent in the open and closed arms of the maze during a 7-minute test. The percentage of time spent in the open arms relative to the total time spent in the maze was calculated for each animal. Less time spent in the open arms is indicative of elevated anxiety-like behavior.

Open Field Test

White plastic open field arenas (50x50x40cm) were evenly illuminated in a dimly lit room (~20Lux). Mice were acclimated to the testing room for 20-minutes, and then placed in the open field arena. Locomotor activity and time spent in center of the arena were recorded during a 7-minute test using the video-tracking system, Ethovision. Using the tracking system, the arena was divided into three zones (each ~1/3 of total surface area of 2,500 in^2); outer ring, middle ring, and the center (Figure 3b). Less time spent in the center of the arena, or more time spent in the outer zone, are indicative of anxiety-like behavior.

SUPPLEMENTAL RESULTS

NIH motivational data

In addition to latency, total time spent drinking was analyzed in the novelty induced hypophagia task during both home cage and novel cage testing. Similar trends were found at both early adolescence and adulthood (**Supplementary Figure 2**). During early adolescence, a significant main effect of day, with animals drinking less in the novel cage ($F_{(7,60)}$=41.979, $p <$ 0.000), as well as a significant sex x rearing condition interaction ($F_{(1,67)}$ = 4.479, p = 0.038), were observed (**Supplementary Figure 2a**). In adult mice, a main effect of rearing condition ($F_{(7,47)}$ = 7.564, p = 0.007) and day ($F_{(7,74)}$ = 6.477, p = 0.013) were found, with ELS animals drinking milk for less time than control animals, and all animals showing a decreased drinking duration in the novel cage. Additionally, a sex by rearing condition interaction was observed, ($F_{(1,81)}$=8.347, p = 0.005). No differences were found between groups in the home cage task phase; but in the novel cage, a significant effect between groups was observed ($F_{(3, 37)}$ = 3.456, p = 0.026), with ELS females drinking for a shorter duration than control females (p = 0.044) (**Supplementary Figure 2b**).

Home cage behavior stats

A general linear models (GLM) repeated measures ANOVA was carried out to test for within subject's effects of hour, as well as interaction between hour, rearing condition, and sex. Between subjects effects of rearing condition and sex were also assessed in this analysis. Significant main effects and interactions from the GLM repeated measures ANOVA were followed up with independent ANOVA's for each hour of behavior and follow-up t-tests for each hour epoch that reached significance with corrections for multiple tests (Tukey's LSD) as well as rearing effects by sex.

Drinking

For drinking behavior, a significant within-subjects effect of hour ($F_{(23,1242)} = 35.680$, $p < 0.001$), as well as hour x condition interaction ($F_{(23,1242)} = 3.481$, $p < 0.001$) were found, with significant variation in drinking behavior over the circadian cycle (**Supplemental Figure 3a**). Investigating between subjects effects, a marginal effect of sex ($F_{(1,54)} = 3.812$, $p = 0.056$) and sex x condition interaction ($F_{(1,54)}$ 3.039, $p = 0.087$) were found, but no main effect of rearing condition ($F_{(1,54)} = 1.712$, $p = .196$). No significant effect of rearing condition was found for drinking behavior in either female ($F_{(1,29)} = 0.118$, $p = 0.734$) or male ($F_{(1,25)} = 3.760$, $p = 0.064$) mice.

Eating

For eating behavior (eating directly from the food hopper), there was a significant effect of hour ($F_{(23, 1242)} = 16.779$, $p = 0.001$), with significant variation in the distribution of eating behavior over the circadian cycle (**Supplemental Figure 3b**). No between subjects effects of sex ($F_{(1,54)} = 1.141$, $p = 0.290$), condition ($F_{(1,54)} = 0.193$, $p = 0.662$), or sex x condition ($F_{(1,54)} = 0.510$, $p = 0.478$) were observed as well as no effect of rearing condition in female ($F_{(1,29)} = 0.031$, $p = 0.861$) or male ($F_{(1,25)} = 0.933$, $p = 0.343$) mice.

Eat from hand

For "eat from hand" (e.g. eating food while on the floor), a significant effect of hour ($F_{(23,1242)} = 14.473$, $p < 0.001$), and hour x sex interaction ($F_{(23,1242)} = 2.556$, $p < 0.001$) were found (**Supplemental Figure 3c**). A between subjects main effect of sex ($F_{(1,54)} = 7.896$, $p = 0.007$) and condition ($F(1,54) = 6.142$, $p = 0.016$) were also found, but no sex x condition interaction ($F_{(1,54)} = 0.040$, $p = 0.843$). In follow-up analyses, a significant effect of rearing condition was found for females ($F_{(1,29)} = 5.279$, $p = 0.029$) but not males ($F_{(1,25)} = 1.852$, $p = 0.186$) mice.

Rearing

For rearing behavior, a significant effect of hour ($F_{(23,1242)} = 54.160$, $p < 0.001$), and hour x condition interaction ($F_{(23,1242)} = 5.815$, $p < 0.001$) were found (**Supplemental Figure 3d**). While there was a significant between subjects effect of condition ($F_{(1,54)} = 5.089$, $p = 0.028$), no effect of sex ($F_{(1,54)} = 0.937$, $p = 0.337$) or sex x condition interaction ($F_{(1,54)} = 1.072$, $p = 0.305$) were observed. A significant effect of rearing condition was found in females ($F_{(1,29)} = 5.662$, $p = 0.024$) but not in males ($F_{(1,25)} = 0.718$, $p = 0.405$).

Hanging

For hanging behavior, a significant effect of hour ($F_{(23,1242)} = 39.189$, $p < 0.001$), and hour x condition interaction ($F_{(23,1242)} = 3.742$, $p < 0.001$) were found (**Supplemental Figure 3e**). Despite significant variation in the distribution of hanging behavior over the course of the day, no main effect of sex ($F_{(1,54)} = 2.896$, $p = 0.095$), condition ($F_{(1,54)} = 0.613$, $p = .437$), or sex x condition interaction ($F_{(1,54)} = 1.362$, $p = 0.248$) were found. Further, no effect of rearing condition was found for hanging behavior in female ($F(1,29) = 0.058$, $p = 0.811$) or male ($F(1,25) = 3.033$, $p = 0.094$) mice.

Undefined movements

For undefined movements, a significant effect of hour ($F_{(23,1242)} = 12.104$, $p < 0.001$), and hour x condition interaction ($F_{(23,1242)} = 2.421$, $p < 0.001$) were found (**Supplemental Figure 3f**). However, no main effect of sex ($F_{(1,54)} = 0.564$, $p = 0.456$), condition ($F_{(1,54)} = 0.435$, $p = 0.512$), or sex x condition interaction ($F_{(1,54)} = 0.329$, $p = 0.568$) were observed. In follow-up analyses, no effects of rearing condition were found for female ($F_{(1,29)} = 0.004$, $p = 0.950$) or male mice ($F_{(1,25)} = 0.714$, $p = 0.406$).
